# Genetic and histological analysis intraplacental choriocarcinoma: a case report

**DOI:** 10.1007/s00795-024-00382-3

**Published:** 2024-02-29

**Authors:** Natsuko Takano, Masashi Takamura, Yosuke Mizuno, Yumi Mizuno, Shunsuke Tamaru, Kohei Nakamura, Hiroaki Soma, Takeshi Kajihara

**Affiliations:** 1 Department of Obstetrics and Gynecology, Faculty of Medicine, Saitama Medical University, 38 Morohongo, Moroyama, Iruma-gun, Saitama, Japan; 2 Division of Morphological Science, Biomedical Research Center, Saitama Medical University, Saitama, Japan; 3 Division of Experimental Animal, Biomedical Research Center, Saitama Medical University, Saitama, Japan; 4 Genomics Unit, Keio Cancer Center, Keio University School of Medicine, Tokyo, Japan; 5 Department of Obstetrics and Gynecology, Kumagaya General Hospital, Saitama, Japan

**Keywords:** Intraplacental choriocarcinoma, Epigenetic change, Methylation, DNA mutation

## Abstract

We report on single case of intraplacental choriocarcinoma (IC) coexisting with feto-maternal hemorrhage from our hospital, a rare malignant tumor that occurs in the chorionic villous trophoblast. To investigate genetic and epigenetic changes to the carcinogenesis of IC, we employed cancer gene panel analysis and whole methylation analysis from a recent case of IC. By Short Tandem Repeats analysis, we confirmed that the tumor of present IC was derived from concurrent normal chorionic villous trophoblast cells. No mutation was found in 145 cancer-related genes. Meanwhile, amplification in *MDM2* gene was observed. Furthermore, we observed deferentially methylated CpG sites between tumor and surrounding normal placenta in present IC case. These observations suggest that IC might be arisen as a result of aberrations of methylation rather than of DNA mutations. Further studies are needed to clarify association between aberrant methylation and choriocarcinogenesis.

**Supplementary Information:**

The online version contains supplementary material available at 10.1007/s00795-024-00382-3.

## Introduction

Intraplacental choriocarcinoma (IC) is an uncommon, highly malignant neoplasm that arises from chorionic villous trophoblasts and can occur in a woman following healthy pregnancy. IC carries the normal number of 46 human chromosomes from both one maternal and one paternal copy. Therefore, it is thought to have a different pathogenesis from other cancers. Driscoll first reported choriocarcinoma in a full-term placenta in 1963 [1], and IC has since been reported in approximately one in 50,000 pregnancies [2, 3]. Half were detected by routine macroscopic examination of the placenta after delivery, so it is likely that the exact number of these placental tumors may be unknown [3]. The most common presentation is vaginal bleeding in the mother, but metastases in more complicated cases can produce symptoms like hemoptysis or cough. Fetal death can occur from feto-maternal hemorrhage (FMH), placental abruption, fetal hydrops with anemia, or intrauterine growth restriction [4]. The prognosis of IC is variable, and both mothers and infants have a risk of distant metastases [3]. Therefore, early detection and introduction of chemotherapy regimens are crucial to improve the overall prognosis. However, the molecular mechanism underlying highly migrative and invasive capacity in IC pathogenesis remains unclear.

Savage et al. analyzed whole genome sequencing and methylation analyses of genetic and epigenetic changes to the pathogenesis of IC. They demonstrated that aberrations of methylation of IC during development, rather than DNA mutations [5]. Since then, there are no similar reports about investigation of molecular mechanism of IC. Here, we have investigated genetic and epigenetic changes in our case of IC using whole genome sequencing and methylation analysis.

## Case presentation

Fetal growth retardation (− 1.7 SD) was detected in a 32-year-old Japanese woman, gravida 7, para 1, by routine clinical check-up at 32 weeks of gestation. Routine fetal cardiotocography revealed a sinusoidal pattern at 35 weeks, and the patient was transferred to our hospital due to suspected fetal anemia. The peak systolic flow velocity in the middle cerebral artery was 92.6 cm/s, comparable with fetal anemia, and a male baby weighing 1871 g was delivered by emergency cesarean section. The Apgar scores were one and five, and the infant was severely anemic with a hemoglobin concentration of 3.7 g/dL. He was intubated, transferred to our Neonatal Intensive Care Unit (NICU), and responded well after a blood transfusion. The infant remained hospitalized for 54 days, and he showed no sign of metastasis in MRI and CT scans.

The maternal serum HbF was 5.5%, compatible with anemia of infant due to FMH, and maternal serum AFP levels was 6061 ng/ml. Maternal MRI and CT scans were unremarkable, and the serum hCG levels were 25 ng/ml and 0.10 ng/ml at 21 and 66 days postpartum, respectively. There has been no clinical evidence of recurrence in either the mother or child in over two years.

## Methods

### Preparation of histopathological specimens

Formalin-fixed paraffin-embedded (FFPE) tissue sections was prepared from the semi-thin section on a slide glass with the pathological block tissue prepared at the time of surgery. The tissue sections were prepared from different block obtained from a patient that include only normal tissue or include mostly tumor tissue. As a control, early gestation villous tissue and full-term villous tissue were prepared during surgery from another patient with the same methods.

### Extraction of genomic DNA

Genomic DNA was extracted from FFPE tissue sections of tumor specimens using Maxwell^®^ RSC DNA FFPE Kit (Promega, Madison, USA) or QIAamp DNA FFPE tissue kit ( QIAGEN, Germany) according to the manufacturer’s instructions.

### Bisulfite treatment and methylation array

EZ DNA Methylation Kit was used for bisulfite treatment of genomic DNA. The bisulfite-treated DNA was labeled with fluorescent dye using Infinium Methylation EPIC Bead Chip Kit, and loaded on Infinium Methylation EPIC Bead Chip. GenomeStudio was used for methylation pattern analysis. DNA methylation level was analyzed with Illumina DiffScore referring GenemeStudio Methylation Module v1.8 User Guide (Illumina), and markers which showed top 50 and bottom 50 DiffScore between sample tumor and surrounding normal placenta was extracted. A clustering analysis was performed and a heatmap was drawn with above extracted 100 markers. Bisulfite treatment and methylation array analysis were performed by RIKEN genesis Co., Ltd.

### STR (short tandem repeats) analysis

STR analysis was performed using AmpFLSTR™ Identifiler™ Plus PCR Amplification Kit (ThermoFisherScientific) according to the manufactural protocol. Briefly, 1.6 ng of genomic DNA purified from IC tumor tissue or its surrounded normal placental tissue were mixed with primer set and PCR master mix and subjected by PCR thermal program. PCR products were electrophoresed by DNA sequencer DNA3500 Genetic Analyzer (Applied Biosystems). Fragment analysis was performed using GeneMapper fragment analysis software (ThermoFisherScientific) and repeat numbers of each STR marker in IC tissue and surrounded normal placental tissue were compared.

### Next generation sceequencing (NGS)

Genomic testing was performed in-house using the PleSSision testing platform (Keio University Hospital, Tokyo, Japan). Libraries were generated from 50 ng of DNA per sample using the Human Comprehensive Cancer Panel, SSEL PreCap Custom Tier2, 6Hybs, SureSelectXT Low Input Reagent Kit, and SureSelectXT HS-LI Enzymatic Frag Kit (Agilent Technologies) and the library quality was assessed using the Agilent High Sensitivity D1000 ScreenTape (Agilent Technologies). Capture sequencing was performed using a 145 cancer-related gene panel (Supplemental Table S1). The enriched libraries were sequenced with a paired-end (150 bp × 2) sequencing method using the NextSeq platform (Illumina, San Diego, CA, USA). Genome annotation and curation for analyzing the sequencing data were performed using an original bioinformatics pipeline called GenomeJack (Mitsubishi Space Software, Tokyo, Japan; http://genomejack.net/english/index.html), in which mapping of the NGS reads to the human reference genome (UCSC human genome 19) was performed using the Burrows–Wheeler Aligner [6], and the reads were realigned with ABRA [7]. For identification of single-nucleotide variants (SNVs), SAMtools was used to pile up the sequencing reads, and defective SNVs that showed conflict between pairwise reads were abandoned [8]. The criteria for mutations were as follows: the noise distributions arising from random sequencing errors were determined. Each mutation was evaluated using a binomial test (*p* < 0.05) to reduce random sequencing errors. We called somatic mutations by comparing the number of mismatch bases in the tumors with those in the normal controls by using the Fisher exact test (*p* < 0.001). The copy number of each gene was calculated as the median value of all the sequencing reads covering the target genes and compared with the median value of the control samples. We identified cancer-specific somatic gene alterations, such as SNVs, insertions/deletions (Indels), and CNAs. All the detected gene alterations in 145 cancer-related genes were annotated and curated using the COSMIC (https://cancer.sanger.ac.uk/cosmic), ClinVar (https://www.ncbi.nlm.nih.gov/clinvar/), CIViC (https://civicdb.org/home), SnpEff24, and Clinical Knowledgebase (CKB) databases (https://ckb.jax.org/).

## Results

### Pathological findings

**Fig. 1:**
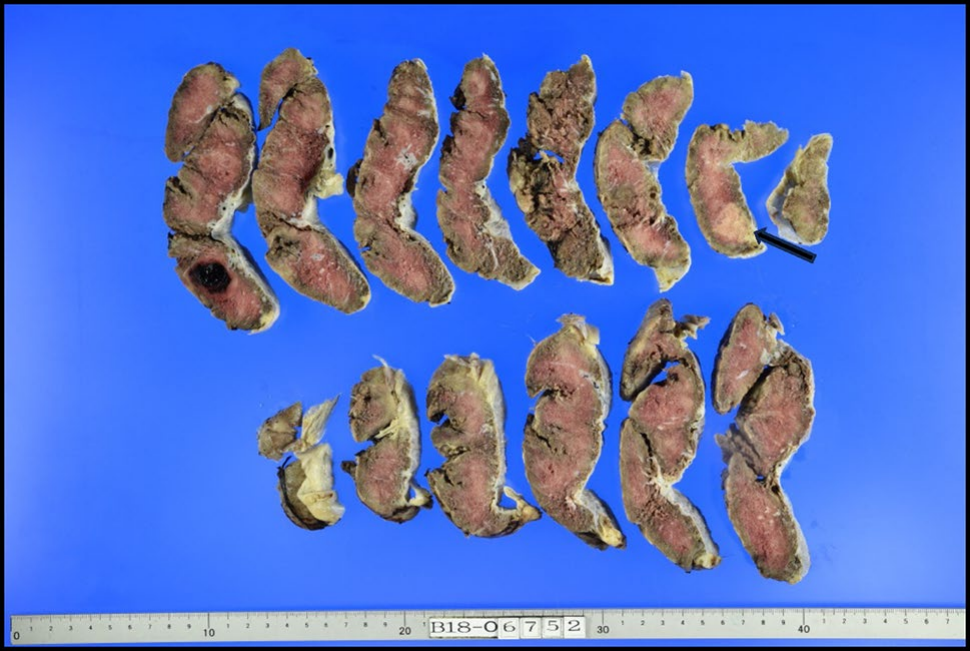
Macroscopic examination of the placenta demonstrated a well-delineated single 2 cm yellow-white area (arrow) on the cut section

**Fig. 2:**
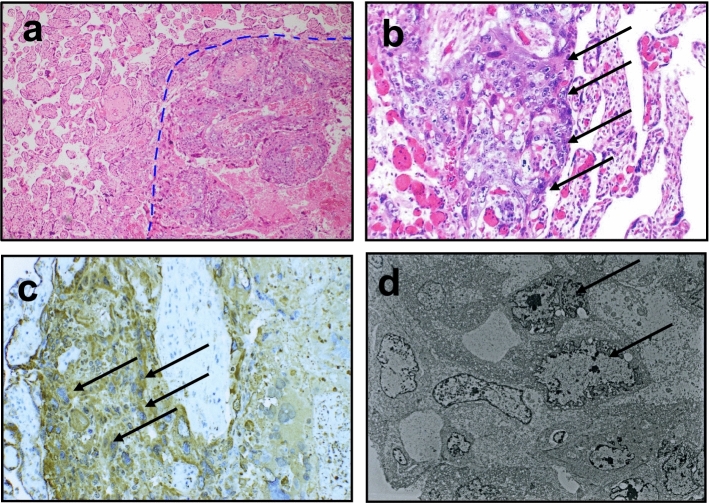
Pathological findings of the intraplacental choriocarcinoma. **a** section of tumor (right) and surrounding normal placenta (left) with a 4 × objective. **b** choriocarcinoma with a biphasic proliferation of atypical and mitotically active cytotrophoblast and syncytiotrophoblast cells (arrows). The neoplasm was restricted to the placenta and did not infiltrate the maternal basal plate with a 20 × objective. **c** Immunohistochemical features of placental choriocarcinoma villi: some carcinoma cells were surrounded by a normal villous mass as revealed by hCG staining (arrows) with a 20 × objective. **d** With electron microscopic observation, these malignant trophoblastic cells were shown to possess large irregular nuclei (arrows) intruding into normal trophoblastic villi

The delivered placenta showed a yellow 2 cm node and a small area of choriocarcinoma (Fig. 1). Microscopic examination showed a choriocarcinoma with a biphasic proliferation of atypical and mitotically active cytotrophoblast and syncytiotrophoblast cells. The neoplasm was restricted to the placenta and did not infiltrate the maternal basal plate (Fig. 2a, b). The placental choriocarcinoma villi were examined, and some carcinomic cell, surrounded by a normal villous mass, expressed hCG by immunohistochemical staining (Fig. 2c). When these malignant trophoblastic cells were observed by an electron microscope, there were shown to have large irregular nuclei intruding into the normal trophoblastic villi (Fig. 2d).

### STR (short tandem repeats) analysis

**Fig. 3:**
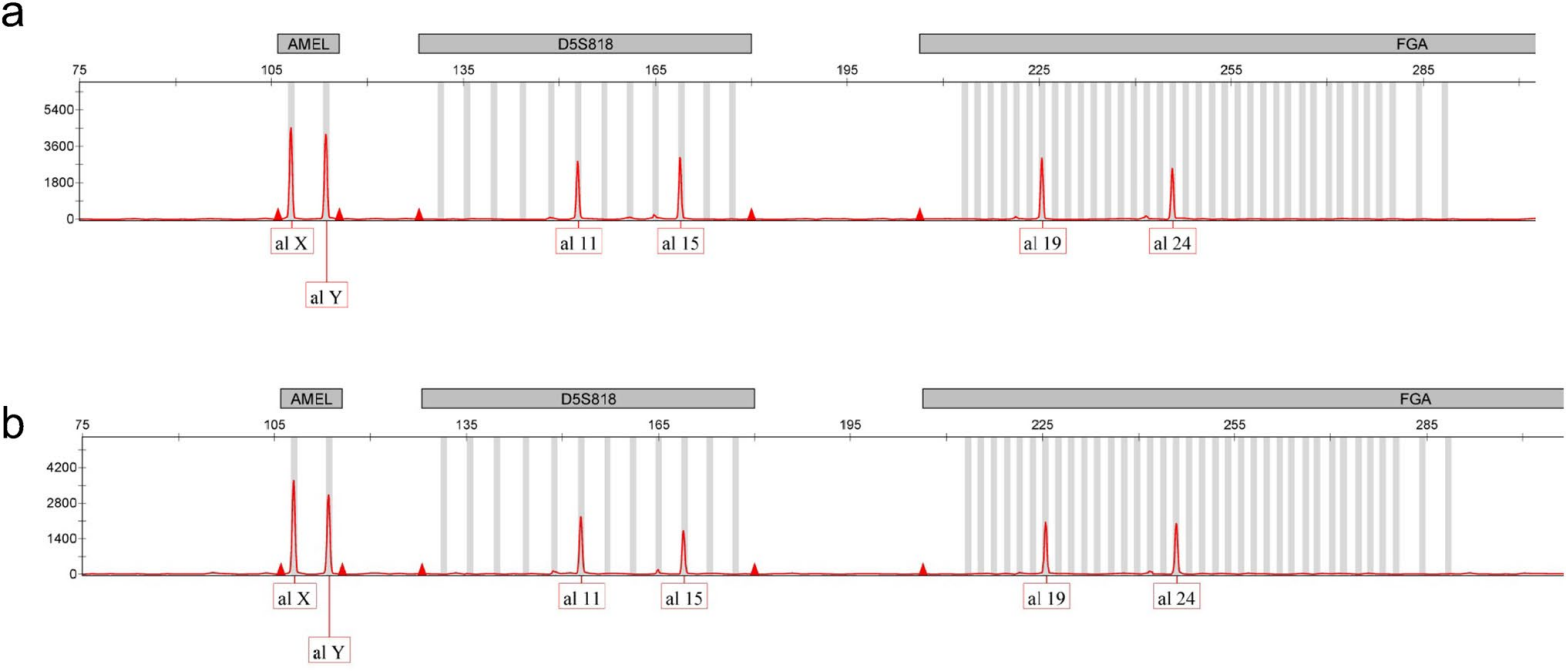
STR analysis. Autosomal sixteen loci in **a** the tumor tissue and **b** those of surrounded normal placental tissue were compared. Three of those loci were shown in Fig. 3

To investigate the origin of IC, we performed DNA STR analysis of IC tumor tissue and normal placental tissue of this case. The result of the STR analysis is shown in Fig. 3. All of analyzed STR alleles in the IC tissue is identical to those of surrounded normal placental tissue, confirming that the IC was derived from concurrent normal chorionic villous trophoblast cells.

### Cancer gene panel analysis using NGS

**Fig. 4:**
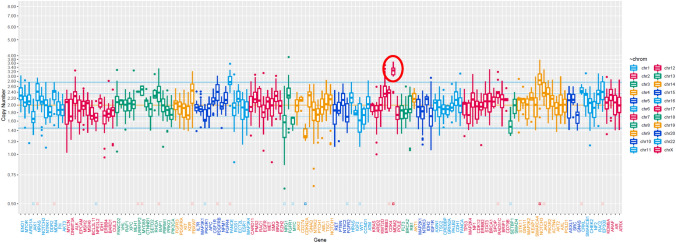
Cancer gene panel analysis. The horizontal axis indicates the genes examined and the vertical axis indicates the copy number. Genetic sequencing identified amplification of MDM2. The circled mark to MDM2

Targeted next-generation sequencing of the patient’s trophoblastic tumor tissue was performed using an in-house assay. No somatic pathogenic mutation of 145 cancer-related genes was observed. Meanwhile, *MDM2* amplification (estimated copy number, 4) was found Fig. 4.

### Whole methylation analysis

**Fig. 5:**
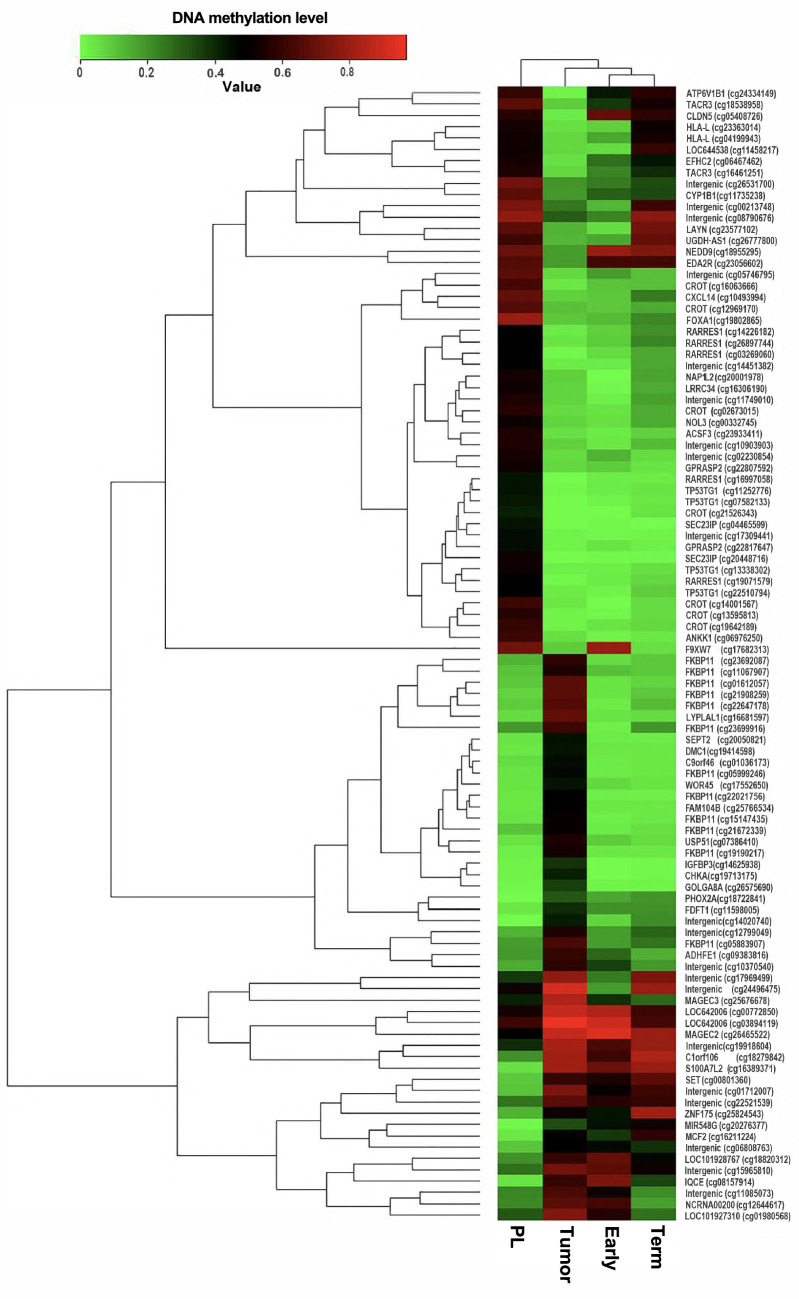
Heatmap of DNA methylation level. Methylation level of each gene markers was measured by Illumina Infinium Methylation EPIC Bead Chip. 50 hyper methylated and 50 hypo methylated Markers top 50 and bottom 50 DiffScore between sample PL and Tumor were extracted. 2D-hierarchical clustering was performed and gene markers and four samples were sorted according to the results of clustering analysis. Early; early gestation villous tissue, Term; full-term villous tissue

We performed DNA Methylation analysis with Infinium Methylation EPIC Bead Chip (Illumina), and a heat map of genes with deferentially methylated CpG sites between tumor (Tumor) and surrounding normal placenta (PL) or unrelated early (Early) and term (Term) placenta samples are shown in Fig. 5. In this figure, 50 top genes with the highest methylated CpG sites and top 50 genes with the most hypo-methylated CpG sites between those samples are filtered, then followed by clustering analysis, and deferentially methylated levels were visualized with a tree-view heat map. By this visualization, genes which have deferentially methylated CpG sites between tumor and surrounding normal placenta are recognized Fig. 5.

## Discussion

Since Driscoll first reported about a case of placental choriocarcinoma in 1963 [1], over 60 cases have been reported [3]. The most common presentation is the vaginal bleeding but the symptoms related metastases like hemoptysis or cough have been reported [2]. Fetal and perinatal reported complications include FMH, placental abruption, fetal hydrops, fetal anemia, intrauterine growth restriction, and fetal death. However, the majority of cases were clinically uneventful and the diagnosis of IC was established on pathological examination of the placenta. Our case was diagnosed by pathological examination of placenta to investigate the cause of fetal anemia. On macroscopically, the lesions of choriocarcinoma usually is not visible and could be mistaken as infarction or hematoma. We, therefore, would like to recommend thorough gross and microscopic examination of placenta, especially in case of FMT, unknown fetal anemia, and abnormal obstetrics events, including premature delivery, still birth, and infantile growth restriction.

Jiao et al. have developed a management algorithm for IC [3]. For a case without maternal or infant metastases and with normal hCG levels, they advise lifelong hCG surveillance without chemotherapy. Our case had no signs of metastasis and low levels of serum hCG. Using Jiao’s algorithm, we administered no chemotherapy, and continued clinical surveillance of the patient’s serum hCG. There has been no clinical evidence of recurrence in either the mother or child in over two years.

Between gestational choriocarcinoma and nongestational choriocarcinoma have dramatically different clinical behavior, sensitivity to chemotherapy, and prognosis. Therefore, distinguishing these tumor subtypes is extremely important [9], but it is difficult to distinguish between those two choriocarcinomas by clinical and pathological examinations [10]. In our case, STR analysis was performed to distinguish gestational and nongestational choriocarcinomas. Examined STR alleles of the IC tissue were identical to those of surrounded normal placental tissue, suggesting that the IC was derived from the concurrent pregnancy.

Savage et al. have used whole sequencing and methylation analysis from a single case to investigate the potential contribution of genetic and epigenetic changes to the pathogenesis of IC. While the tumor had a low level of mutation, driver mutation, or oncogene activity, it had a different methylation profile than found in the mature placenta. They conclude that IC is likely to arise as result of aberrations of methylation rather than of DNA mutations [5]. Likewise, we observed deferentially methylated CpG sites between tumor and surrounding normal placenta in our case.

There are several reports about correlation between gestational trophoblastic disease and epigenetic alternations at the DNA methylation level. The present study demonstrated that the pattern of methylation is markedly different between the tumor and surrounding normal placenta. Interestingly, some genes that were identified as top 50 hyper or hypo methylation by our whole methylation analysis had already been reported in association between aberrant methylation of their promoters and choriocarcinogenesis. Sasagawa et al. demonstrated that on Soluble Fms-like tyrosine kinase-1(sFLT1) promoter region in choriocarcinoma cell lines and a choriocarcinoma tissue, and it caused suppression of sFLT1 production. Furthermore, the stable expression of sFLT1 in choriocarcinoma cells inhibited cancer progression in vivo [11]. Those findings were consistent with our present study (Fig. 5). On the other hand, Huebner et al. demonstrated that choriocarcinoma cell lines showed a hypermethylation of the Retinoic acid receptor responder 1 (RARRES1) promoter, which was identified as an important tumor suppressor gene [12, 13], and correlated with a reduced *RARRES1* expression [14]. However, our present study demonstrated that the promoter region of *RARRES1* in tumor was hypomethylated in comparison with surrounded normal tissue. There are some possibilities that our findings are not consistent with previous studies. First, although analysis of previous reports was performed using choriocarcinoma cell lines, we analyzed using IC tissues. Second, we performed whole methylation analysis using only one case. Therefore, it is difficult to draw conclusion based on analysis from our single case.

By our cancer gene panel analysis using NGS, no mutations were found in 145 cancer-related gene. Meanwhile, *MDM2* amplification was observed. MDM2 targets p53 for ubiquitin-dependent proteasomal degradation and recruiting transcriptional co-repressors of p53 [15]. Overexpression of *MDM2* is functionally similar to mutation in p53 gene. It has been reported that more than 17% of human tumor exhibited *MDM2* gene amplification [16]. To our knowledge, there is no report about association between *MDM2* alternation and gestational trophoblastic disease. However, further study needs to clarify corresponding between *MDM2* amplification and choriocarcinogenesis.

## Conclusion

In this article, we report single case of IC coexisting with feto-maternal hemorrhage from our hospital. We have investigated genetic and epigenetic changes using whole genome sequencing and methylation analysis from our case of IC. By STR analysis, we confirmed that the present IC was derived from concurrent normal chorionic villous trophoblast cells. And we observed that there was no mutation of 145 cancer-related gene. Meanwhile, *MDM2* amplification was observed. Furthermore, we observed deferentially methylated CpG sites between tumor and surrounding normal placenta in present IC case. These observations suggest that IC might be arisen as result of aberrations of methylation rather than of DNA mutations. Further studies are need to clarify association between aberrant methylation and choriocarcinogenesis.

### Supplementary Information

Below is the link to the electronic supplementary material.

Supplementary file1 (DOC 90 KB)
