# Supplementary material for: Genetic and histological analysis intraplacental choriocarcinoma: a case report
Source: Med Mol Morphol. 2024 Feb 29;57(2):147–54. doi: 10.1007/s00795-024-00382-3 (PMC11128402; doi:10.1007/s00795-024-00382-3)
Supplement: Supplementary file 1 — Supplementary file1 (DOC 90 KB) [file 795_2024_382_MOESM1_ESM.doc]

Supplementary Table 1. List of 145 Genes Analyzed in the Targeted-Gene Panel

| *ABL1* | *ACTN4* | *AKT1* | *AKT2* | *AKT3* | *ALK* | *APC* | *AR* |
| --- | --- | --- | --- | --- | --- | --- | --- |
| *ARAF* | *ARID1A* | *ARID2* | *ASXL1* | *ATM* | *ATRX* | *AXIN1* | *AXL* |
| *BAP1* | *BARD1* | *BCL2L11* | *BRAF* | *BRCA1* | *BRCA2* | *BRIP1* | *CARD11* |
| *CCND1* | *CD274* | *CD79B* | *CDH1* | *CDK12* | *CDK4* | *CDKN2A* | *CHEK2* |
| *CREBBP* | *CRKL* | *CSF1R* | *CTNNB1* | *CUL3* | *DDR2* | *DNMT3A* | *ECT2L* |
| *EGFR* | *ENO1* | *EP300* | *EPCAM* | *ERBB2* | *ERBB3* | *ERBB4* | *ESR1* |
| *EZH2* | *FANCA* | *FANCD2* | *FANCE* | *FBXW7* | *FGFR1* | *FGFR2* | *FGFR3* |
| *FGFR4* | *FH* | *FLT3* | *GNA11* | *GNAQ* | *GNAS* | *GRIN2A* | *HRAS* |
| *IDH1* | *IDH2* | *IGF1R* | *IGF2* | *IL7R* | *JAK1* | *JAK2* | *JAK3* |
| *KDM6A* | *KDR* | *KEAP1* | *KIT* | *KMT2D* | *KRAS* | *MAP2K1* | *MAP2K2* |
| *MAP2K4* | *MAP3K1* | *MAP3K4* | *MDM2* | *MDM4* | *MED12* | *MET* | *MLH1* |
| *MSH2* | *MSH6* | *MTOR* | *MYC* | *MYCN* | *MYD88* | *NF1* | *NF2* |
| *NFE2L2* | *NOTCH1* | *NOTCH2* | *NOTCH3* | *NPM1* | *NRAS* | *NRG1* | *NT5C2* |
| *NTRK1* | *NTRK2* | *NTRK3* | *PALB2* | *PBRM1* | *PDGFRA* | *PDGFRB* | *PIK3CA* |
| *PIK3RI* | *PIK3R2* | *PMS2* | *POLD1* | *POLE* | *PRKCI* | *PTCH1* | *PTEN* |
| *RAC1* | *RAC2* | *RAD51C* | *RAF1* | *RB1* | *RET* | *RHOA* | *ROS1* |
| *SETBP1* | *SETD2* | *SMAD4* | *SMARCA4* | *SMARCB1* | *SMO* | *SPOP* | *SRC* |
| *STAT3* | *STK11* | *TERT* | *TP53* | *TSC1* | *TSC2* | *VHL* | *WT1* |
| *XPC* |  |  |  |  |  |  |  |
